# Correlates of Sleep Disturbance among Older Adults with Mild Cognitive Impairment: A Cross-Sectional Study

**DOI:** 10.3390/ijerph17134862

**Published:** 2020-07-06

**Authors:** Dan Song, Doris S. F. Yu, Qiuhua Sun, Guijuan He

**Affiliations:** 1 School of Nursing, Zhejiang Chinese Medical University, Hangzhou 310053, China; sunqiuhua@zcmu.edu.cn (Q.S.); 19841015@zcmu.edu.cn (G.H.); 2 The School of Nursing, the University of Hong Kong, Hong Kong, China; dyu1@hku.hk

**Keywords:** cross-sectional study, correlates, mild cognitive impairment, sleep

## Abstract

Individuals with mild cognitive impairment (MCI) are at high risk for dementia development. Sleep disturbance is often overlooked in MCI, although it is an important risk factor of cognitive decline. In the absence of a cure for dementia, managing the risk factors of cognitive decline in MCI is likely to delay disease progression. To develop interventions for sleep disturbance in MCI, its related factors should be explored. This study aimed to identify and compare the correlates of sleep disturbance in older adults with MCI and those in cognitively healthy older adults. A comparative cross-sectional study was adopted. Data were obtained from 219 Chinese community-dwelling older adults (female: 70.3%), which consisted of 127 older adults with MCI and 92 age-matched cognitively healthy controls. The candidate correlates of sleep disturbance included socio-demographic correlates, health-related factors, lifestyle-related factors and psychological factor. Descriptive, correlational and regression statistics were used for data analysis. The prevalence of sleep disturbance in MCI was 70.1% compared to that of 56.5% in cognitively healthy controls (*p* < 0.001). The multivariate analysis indicated that, in participants with MCI, depressive symptoms (Beta = 0.297, *p* = 0.001), comorbidity burden (Beta = 0.215, *p* = 0.012) and physical activity (Beta = −0.297, *p* = 0.001) were associated with sleep disturbance. However, in the cognitively healthy controls, only depressive symptoms (Beta = 0.264, *p* = 0.028) and comorbidity burden (Beta = 0.361, *p* = 0.002) were associated with sleep disturbance. This finding highlights that sleep disturbance is sufficiently prominent to warrant evaluation and management in older adults with MCI. Furthermore, the findings elucidate several important areas to target in interventions aimed at promoting sleep in individuals with MCI.

## 1. Introduction

The dramatic growth of the worldwide aging population implies an imminent increase in the number of old people afflicted with cognitive impairment. Mild cognitive impairment (MCI) is a precursor of dementia; afflicted individuals show cognitive impairment beyond those seen in their normal age but does not reach dementia [1]. The prevalence of MCI ranges from 16–22.2% worldwide [2,3,4]. Individuals with MCI represent a high-risk group for dementia development [1]. It is estimated that 10% of MCI patients develop dementia annually, whereas the annual progression rate to dementia is around 1–3% in the overall older adults [5,6]. The high prevalence, progression rate to dementia and considerable healthcare expenditure have made MCI as a major public health concern.

In the absence of a cure for dementia, managing the risk factors of cognitive decline in MCI has a high potential to delay the disease progression. Individuals with MCI commonly experience neuropsychiatric symptoms that mediate the disease deterioration in cognitive impairment. The most commonly studied neuropsychiatric symptoms in MCI are depression, apathy and anxiety [7]. Sleep disturbance, however, has been relatively overlooked and understudied in MCI. A recent systematic review on neuropsychiatric symptoms in MCI found limited information regarding the pattern of sleep disturbance, as the vast majority of the selected studies utilised the 10-item version of the Neuropsychiatric Inventory (NPI-10), which does not include a subscale for sleep disturbance [8]. Studies that specifically investigated the sleep condition in MCI revealed that sleep disturbance can be prevalent in the MCI population with an estimated prevalence of 38.3–63%, which is more common than that in the cognitively healthy counterparts [9,10,11]. Sleep disturbance induces fatigue, disturb daily function and reduce quality of life among older adults [12,13]. Furthermore, it is a strong risk factor for cognitive decline in MCI. In a longitudinal study with four year follow up, sleep disturbance doubled the risk of cognitive decline in community-dwelling healthy older adults, and reduced the chance of reversion to normal cognition by 31% in those with MCI [14]. Therefore, more attention to sleep disturbance in the context of care for individuals with MCI is warranted.

In the current literature, there are no intervention studies targeting sleep disturbance for individuals with MCI. A key step in designing interventions for sleep disturbance in MCI is to identify the factors related to sleep disturbance in this population. However, in the literature, few studies have explored the factors associated with sleep disturbance among individuals with MCI.

Studies conducted among older adults with or without cognitive impairment indicated that many health-related, psychosocial and lifestyle factors may be associated with sleep disturbance.

Among the health-related factors, multimorbidity, low functional and health perception may impair sleep quality by inducing remarkable physical and psychological burden [15,16]. Among the lifestyle factors, being physically active relieves stress level, benefits sleep physiology and is consistently associated with better sleep quality in the older population [17]. Moreover, alcohol consumption disrupts sleep architecture and negatively influences sleep quality [18]. As for the psychosocial factors, various socio-demographic factors, including old age, being female, low education level, lower income, living alone, being single and experience of stressful life events may expose older adults to high sleep disturbance [19], although the associations are often inconsistent across studies. Depressed mood is strongly correlated with sleep disturbance. This may be due to the fact that individual with depression often have intrusive thoughts and hyper arousal symptoms, thereby making it difficult for them to fall asleep and to stay asleep [20].

As one of the neuropsychiatric symptoms in MCI, sleep disturbance may follow a different pathway in its manifestation. Therefore, factors associated with sleep disturbance in individuals with MCI may be different from other members of the population. A comprehensive investigation of the correlates of sleep disturbance in this distinct population will add new knowledge to the existing literature.

The purpose of the present study was (1) to investigate the prevalence of sleep disturbance among individuals with MCI and compare the prevalence to that in the cognitively healthy controls; (2) to comprehensively investigate the correlates of sleep disturbance among individuals with MCI and compare the results in MCI to those in the cognitively healthy controls. The research hypothesis was that (1) there is a high prevalence of sleep disturbance a high prevalence among individuals with MCI, and the prevalence is higher than that in the cognitively healthy controls (2) various socio-demographic factors, health-related factors, lifestyle-related factors and psychological factors were associated with sleep disturbance among individuals with MCI, and the identified correlates of sleep disturbance in MCI were different from those in the cognitively healthy controls. The findings will elucidate the development of healthcare services and caregiver strategies that can improve sleep quality and delay disease deterioration for this distinct clinical cohort.

## 2. Materials and Methods

### 2.1. Ethics Statement

This study was approved by the Survey and Behavioral Research Ethics Committee of the Chinese University of Hong Kong (No. SBREC-20160602). Consent forms were obtained from the participants before the data collection. The participants were assured of the confidentiality of their data.

### 2.2. Study Design and Sample

This study followed the Strengthening the Reporting of Observational studies in Epidemiology (STROBE) guidelines for reporting. A cross-sectional comparative study design was used. Participants were community-dwelling older adults with MCI. They are aged 60 years old or older. A convenient sample of 127 individuals with MCI was recruited, as well as 92 aged-matched cognitively healthy counterparts. The Montreal Cognitive Assessment (Chinese version, MoCA-C) was used to identify potential subjects with MCI [21]. By using a cut-off score of 19 and 26, the MoCA-C provides optimal sensitivity and specificity in differentiating individuals with MCI from those with dementia (sensitivity: 93.2%; specificity: 71.7%) and intact cognitive function (sensitivity: 92.4%; specificity: 88.4%) [22]. The influence of education on cognitive function was adjusted by adding one point to those with less than six years of education [23].

Participants were excluded if they met the following criteria: (1) individuals who score below 19 on MoCA-C or have a diagnosis of any type of dementia; (2) individuals who have any serious neurological disorders that influence the cognition (e.g., stroke, Parkinson’s disease and head damage); (3) individuals who have impaired hearing or vision that may inhibit them from giving consent and answering the questionnaires.

Regarding sample size estimation, researchers conservatively estimated a medium effect size (*r* = 0.25) of the relationship between sleep quality and the group of candidate correlates [24]. Fourteen independent variables were considered to be included into the multivariate analysis. In this study, two multivariate regression models were built to examine and compare the correlates of sleep disturbance in MCI group and cognitively healthy controls. Thus, a minimum of 86 participants in each group was required in this study.

### 2.3. Data Collection and Measurement

Data collection took place in a public community healthcare center in the city of Hangzhou, Southeast China from June 2016 to May 2017. Participants for this study were recruited via the following: (1) study posters attached with the contact information of the researchers; (2) health talks held at the community healthcare center; (3) and word of mouth by the researchers. Individuals who show interest were invited for an in-person interview to screen for study eligibility. Three research nurses consented and collected data from the eligible participants. Data were collected using paper and pencil method. A number of validated tools were used to measure the criterion variable of sleep quality and candidate correlates including biological (chronic disease burden, functional status and self-health perception), lifestyle (physical activity and alcohol), socio-demographic (age, gender, marriage, education, income, residence and experience of stressful life events) and psychological (depressed mood) correlates.

Sleep quality was measured by the Pittsburgh sleep quality index (PSQI). It provides a global sleep quality score (maximum = 21) based on seven components (maximum sub-scale score = 3) including sleep quality, latency, duration, efficiency, disturbance, use of sleep medication, and daytime dysfunction due to poor sleep quality [25]. The PSQI displays good reliability with Cronbach’s α of 0.83 [25]. A global score ≥ 6 yielding a diagnostic sensitivity of 90% and specificity of 67% in differentiating good and poor sleepers among Chinese population [26]. The Cronbach’s alpha of PSQI in this study was 0.736.

The burden of chronic diseases was quantified by the Charlson comorbidity index [27]. This well-established scale comprises 19 coexisting medical conditions with corresponding weights, with a higher score indicating greater burden of chronic disease. Health perception was indicated by EuroQoL visual analogue scale (EQ-VAS) (0–100) [28], with high scores indicating high self-rated health status. Functional status was assessed by the 10-item functional activities questionnaire (FAQ), which evaluates complex functional and social behaviors of older adults that are probably impaired during early cognitive decline stage [29]. FAQ is considered as more sensitive than the Lawton and Brody’s instrumental activities of daily living (IADL) scale (sensitivity: 85% vs. 57%) in detecting functional impairment in those with early cognitive impairment [29].

A socio-demographic sheet was designed to obtain the socio-demographic data, including age, gender, education, marital status, living condition, income, and experience of stressful life events. A list of major life events relevant to Chinese older adults developed by Yu et al. Ref. [30] was used to determine the number of major stressful life events over the past year. These events included the following: severe illness or injury, discord with spouse or in-laws, change in financial situation, hospitalization or moving out and death of spouse, close friends or other family members. The individual’s mood status was assessed by the 30-item geriatric depression scale (GDS). The scale results of using dichotomous questions presented a total score ranging from 0 to 30, with high scores representing highly depressive symptoms. Good internal consistency (Cronbach’s α = 0.846) and construct validity of Chinese version of GDS are also reported [31]. The Cronbach’s alpha of GDS in this study was 0.784.

Physical activity level was measured by the international physical activity questionnaires short version (IPAQ-SF). The IPAQ-SF classifies the level of physical activity into three groups: low, moderate and high. Criterion validity was established by the significant correlation with the pedometer-measured steps [32]. Alcohol consumption measured by standard drinks (10 grams of pure alcohol) per week was also collected.

### 2.4. Statistical Analysis

Statistical analysis was performed with SPSS version 22. Correlation analysis was conducted to examine significant bivariate associations and assess for multi-collinearity between all the dependent and independent variables, using the Pearson’s correlation and Spearman’s Rho for the continuous and ordinal variables, respectively. Multiple linear regression analysis was then conducted to identify the independent correlates of sleep quality both in MCI participants and cognitively healthy controls, so as to compare the significant correlates of sleep disturbance between those with and without cognitive impairment. The significance level α was set at 0.05, and all comparisons were two tailed.

## 3. Results

### 3.1. Characteristics of the Participants

The characteristics of the participants are summarised in Table 1. Compared with the cognitively healthy controls, individuals with MCI were more likely to be female, had lower education level, lower income and more chronic disease burden. They reported more functional difficulties and experienced more depressive symptoms. Individuals with MCI had a higher likelihood of sleep disturbance (70.1%) compared with the controls (56.5%). Among the PSQI subscales, individuals with MCI reported lower sleep quality, longer sleep latency, less sleep duration, more daytime dysfunction and used sleep medications more frequently.

### 3.2. Bivariate Assocaitions between Sleep Quality and Candidate Correlates

For the participants with MCI, bivariate associations between sleep quality and all the candidate correlates are presented in Table 2. Being female (*r* = 0.189, *p* = 0.033), higher burden of chronic diseases (*r* = 0.271, *p* = 0.002), more functional difficulties (*r* = 0.238, *p* < 0.001), lower perceived health status (*r* = −0.278, *p* = 0.002), lower physical activity level (*r* = −0.247, *p* = 0.005) and higher depressive symptoms (*r* = 0.440, *p* < 0.001) were significantly associated with poorer sleep quality.

As for the cognitively healthy controls, higher burden of chronic diseases (*r* = 0.426, *p* < 0.001), lower perceived health status (*r* = −0.336, *p* = 0.001) and higher depressive symptoms (*r* = 0.351, *p* = 0.001) were significantly associated with more sleep disturbance.

### 3.3. Multivariate Correlates of Sleep Disturbance

Table 3 summarized the results of the multivariate correlates of sleep disturbance. For the entire group of participants, hierarchy regression analysis was conducted to explore whether the presence of MCI was associated with a higher likelihood of sleep disturbance. The results showed that the presence of MCI was associated with sleep disturbance after adjusting for the effects of socio-demographic, health-related and lifestyle related factors; however, the significant association disappeared when depressive symptoms was added to the model.

For the participants with MCI, depressive symptoms (Beta = 0.297, *p* = 0.001), comorbidity burden (Beta = 0.215, *p* = 0.012) and physical activity level (Beta = −0.297, *p* = 0.001) were identified as independent correlates of sleep disturbance among individuals with MCI.

As for cognitively healthy controls, only depressive symptoms (Beta = 0.264, *p* = 0.028) and comorbidity burden (Beta = 0.361, *p* = 0.002) were identified as independent correlates of sleep disturbance among cognitively healthy older adults. No other associations were significant.

## 4. Discussion

Study results confirmed the research hypothesis that high prevalence of sleep disturbance exists among older adults with MCI. Seventy percent of the participants with MCI had sleep disturbance, which was significantly higher compared to the cognitively healthy controls (56.5%).

Regarding the correlates of sleep disturbance, the study results confirmed the hypothesis that various factors are associated with sleep disturbance in MCI. Among all the candidate correlates, higher level of depressive symptoms, more chronic disease burden and lower level of physical activity were identified to be significantly correlated with sleep disturbance among older adults with MCI. Furthermore, physical activity is identified as a specific correlate of sleep disturbance for MCI, as the association between physical activity and sleep disturbance disappeared when we also analysed it among the cognitively healthy controls. These findings have substantial implications for clinical practice.

Depressive symptoms were identified as the strongest correlates of sleep disturbance in MCI. In fact, the significant association between the presence of MCI and sleep disturbance disappeared when taking account depressive symptoms in the relationship, suggesting that depressive symptoms may be important mediators of the cognition–sleep relationship [33]. Depressive symptoms are among the most prevalent neuropsychiatric symptoms in MCI [34]. The strong association between depressive symptoms and sleep disturbance alerts the healthcare providers to evaluate both symptoms in MCI. Ignoring either symptom will trap the individuals with MCI in the vicious circle of low mood–poor sleep. The significant role of depressive symptoms in affecting sleep disturbance in MCI also sheds light on the modification of sleep disturbance by adopting evidence-based psychological interventions, including cognitive-behavioural therapy, supportive psychotherapy, problem-solving therapy and interpersonal therapy that can effectively relieve depressive symptoms [35].

The presence of multimorbidity in MCI also increases the likelihood of sleep disturbance, which is consistent with previous studies in general older adults [36]. This may be due to the fact that multimorbidity causes concern over one’s health status, thereby disturbing sleep quality [37]. This finding implies that healthcare providers need to pay more attention to MCI patients with multimorbidity. They may be at higher risk of developing sleep disturbance than those without multimorbidity. The presence of sleep disturbance may in turn worsen the existing medical condition, as it may induce fatigue and low mood, thereby making the management of disease conditions more difficult. Thus, the healthcare providers must be vigilant in their assessment and care of those with MCI and sleep disturbance.

Low level of physical activity was correlated with sleep disturbance in MCI, and this association was stronger in MCI compared with cognitively healthy controls. This may be partly explained by the difference of antioxidant capacity level between MCI patients and cognitively healthy controls. MCI patients show a reduced antioxidant capacity compared with the cognitively healthy controls, which was associated with sleep disturbance in MCI [38]. Exercise demonstrates strong effects on the improvement of antioxidant defenses [39], thereby playing an important role in mediating the effects of oxidative stress on sleep quality in MCI. The significant effect of low level physical activity on sleep disturbance of individuals with MCI highlighted the importance of encouraging regular physical activity in individuals with MCI. To achieve this, healthcare providers should carefully assess the factors that positively or negatively influence their participation in physical activity and help older adults with MCI to overcome barriers to participation, such as fear and lack of confidence. Group-based exercise interventions indicated satisfactory adherence among older adults with MCI [40], as they provide a platform for older adults with MCI to have a sense of social cohesion, thereby promoting physical activity. Such interventions should be applied to older adults with MCI to promote their sleep quality.

### 4.1. Implications

Healthcare professionals across levels of practice settings provide care to individuals with cognitive impairment. Sleep disturbance is relatively overlooked in the population with MCI. This study indicated that sleep disturbance is more prevalent in MCI compared to that in the cognitively healthy controls, thus highlighting that sleep disturbance is prominent enough to warrant evaluation and management among older adults with MCI.

Although interventions that target sleep disturbance in MCI are lacking, the correlates of sleep disturbance identified in this study shed light on the development of interventions aiming at relieving sleep disturbance in MCI. Depressive symptoms, comorbidity burden and being physical inactive were identified as correlates of sleep disturbance in MCI. Therefore, psychotherapies and exercise interventions may be incorporated into nursing practice to improve the sleep quality of individuals with MCI. Encouraging regular physical activity in individuals with MCI is especially important, as being physical inactive was identified to be solely correlated with sleep disturbance in older adults with MCI but not in cognitively healthy controls.

### 4.2. Limitations and Future Lines of Research

The present study has several limitations. Firstly, the cross-sectional design of this study did not allow the determination of the direction of the relationships. A longitudinal study design should be used in future studies and in further testing of the findings. Secondly, the small sample size and convenience sampling limited the generalizability of the study findings. A larger sample size and more stringent sampling method are needed in future studies to enhance the generalizability of the findings. Thirdly, only select socio-demographic, health-related, lifestyle and psychological variables were examined for their associations with sleep disturbance. The impact of other intrapsychic variables and sleep-related beliefs on sleep disturbance requires further exploration to enable more comprehensive examination of the predictive model. Finally, only a subjective sleep measure was used in this study. Other objective measures of sleep should be tested as independent variables in future models when exploring correlates of sleep in MCI.

## 5. Conclusions

The current study added new knowledge to the existing literature regarding the correlates of sleep disturbances in the MCI population. Individuals with MCI who have more depressive symptoms, multimorbidity and sedentary lifestyle were more likely to experience sleep disturbance. The study findings facilitated the identification of the specific group who are at greater risk of developing sleep disturbance and elucidated the development of interventions to promote sleep quality in this clinical cohort.

## Figures and Tables

**Table 1:** Sample characteristics (*n* = 219).

Characteristics	Total (*n* = 219)	MCI (*n* = 127)	Controls (*n* = 92)	*p*
Age	73.76 ± 7.61	74.24 ± 7.15	73.11 ± 8.20	0.280
Gender				0.160
Male	65 (29.7%)	33 (26.0%)	32 (34.8%)	
Female	154 (70.3%)	94 (74.0%)	60 (65.2%)	
Marital status				0.127
Married	269 (72.8%)	96 (75.6%)	71 (77.2%)	
Single	101 (27.3%)	31 (24.4%)	21 (22.8%)	
Education level				<0.001
Less than 6 years’ education	103 (27.8%)	39 (30.7%)	9 (9.8%)	
Above 6 years’ education	267 (72.2%)	88 (69.3%)	83 (90.2%)	
Residence				0.741
Living alone	83 (49.5%)	26 (20.5%)	21 (22.8%)	
Living with others	187 (50.5%)	101 (79.5%)	71 (77.2%)	
Monthly income				0.021
Less than 4000 CNY	233 (63.0%)	85 (66.9%)	50 (54.4%)	
Above 4000 CNY	137 (37.0%)	42 (33.1%)	42 (45.6%)	
No. of major stressful life events				0.749
0	116 (56.9%)	72 (63.1%)	52 (53.9%)	
1–3	88 (43.1%)	55 (36.9%)	40 (46.1%)	
Alcohol, standard drink/week	1.12 ± 2.65	0.95 ± 2.54	1.35 ± 2.78	0.271
Physical activity level				0.601
Low	68 (18.4%)	24 (18.9%)	20 (21.7%)	
Moderate	292 (78.9%)	101 (79.5%)	69 (75.0%)	
Vigorous	10 (2.7%)	2 (1.6%)	3 (3.3%)	
Objective cognition (MoCA)	24.71 ± 2.60	22.93 ± 1.94	27.17 ± 1.14	<0.001
Comorbidity burden (CCI)	1.72 ± 1.07	1.70 ± 0.96	1.74 ± 1.21	0.795
Perceived health status (EQ-VAS)	74.74 ± 10.83	75.46 ± 10.38	73.76 ± 11.41	0.254
Functional status (FAQ)	0.33 ± 0.65	0.45 ± 0.75	0.16 ± 0.43	0.001
Level of depressive symptoms (GDS)	4.51 ± 3.61	5.50 ± 4.05	3.13 ± 2.27	<0.001
Global sleep quality (PSQI total score)	8.00 ± 4.22	8.65 ± 4.31	7.10 ± 3.95	0.007
Sleep quality	1.28 ± 0.62	1.35 ± 0.64	1.18 ± 0.59	0.046
Sleep latency	1.01 ± 1.05	1.17 ± 1.05	0.78 ± 1.01	0.006
Sleep duration	1.74 ± 0.95	1.82 ± 0.87	1.62 ± 1.05	0.126
Sleep efficiency	1.53 ± 1.22	1.57 ± 1.17	1.48 ± 1.29	0.598
Sleep disturbance	1.11 ± 0.45	1.13 ± 0.48	1.08 ± 0.43	0.356
Use of sleep medications	0.55 ± 1.08	0.65 ± 1.15	0.42 ± 0.96	0.132
Daytime dysfunction	0.78 ± 0.84	0.95 ± 0.89	0.53 ± 0.70	<0.001
Presence of sleep disturbance	141 (64.4%)	89 (70.1%)	52 (56.5%)	0.046

Note. CNY: Chinese yuan; CCI: Charlson comorbidity index; EQ-VAS: EuroQol-visual analog scale; GDS: geriatric depression scale; MoCA: Montreal cognitive assessment.

**Table 2:** Association between socio-demographic features, lifestyle factors, health-related, psychological factors, and sleep quality in mild cognitive impairment (MCI, *n* = 129).

	Variable	1	2	3	4	5	6	7	8	9	10	11	12	13	14
1	Global Sleep quality	1.00													
2	Age	0.067	1.00												
3	Gender	0.189 *	−0.318 *	1.00											
4	Income	−0.082	0.264 **	−0.442 **	1.00										
5	Marital status	−0.019	0.235 **	0.128	−0.092	1.00									
6	Living condition	−0.053	−0.166 *	−0.038	0.094	−0.274 **	1.00								
7	Education	−0.022	0.072	−0.267 **	0.365 **	−0.237 **	0.029	1.00							
8	No. of stressful life events	0.051	0.106	−0.099 *	0.125	0.052	0.131	0.067	10.00						
9	Chronic disease burden	0.271 **	0.116	0.048	0.118	0.099	0.048	−0.168	0.176 *	10.00					
10	Alcohol drinking	0.123	−0.007	−0.065	0.007	0.000	0.050	−0.036	−0.017	−0.083	1.00				
11	Functional status	0.312 **	0.198 *	−0.069	0.080	0.020	0.113	−0.032	0.087	0.000	−0.083	1.00			
12	Perceived health status	−0.272 **	0.148	−0.071	−0.015	0.048	−0.011	−0.040	−0.009	−0.008	0.000	−0.181 *	1.00		
13	Physical activity level	−0.247	−0.003	−0.115	−0.007	0.57	−0.076	−0.062	0.0151	0.096	0.008	−0.083	0.167	1.00	
14	Depressive symptoms	0.440 **	−0.025	0.118	−0.050	0.061	−0.034	−0.058	0.095	0.177 *	0.096	0.369 **	−0.393 **	−0.067	1.00

Note: * *p* < 0.05, ** *p* < 0.01

**Table 3:** Multivariate correlates of sleep disturbance (*n* = 219).

Model	B (SE)	Beta	R^2^	Change in R^2^	*p* Value
Model 1: Whole group (*n* = 219)					
Block 1			0.033	0.033	0.007
The presence of MCI	1.548 (0.570)	0.181			0.007
Block 2			0.074	0.039	0.258
The presence of MCI	1.443 (0.601)	0.169			0.017
Age	0.071 (0.041)	0.128			0.084
Female	1.250 (0.723)	0.136			0.085
Income	−0.085 (0.444)	−0.015			0.848
Education	0.034 (0.325)	0.008			0.916
Being single	−0.757 (0.366)	−0.155			0.040
Living alone	−0.767 (0.515)	−0.105			0.138
Number of stressful life events	0.566 (0.463)	0.084			0.222
Block 3			0.313	0.239	<0.001
The presence of MCI	1.383 (0.540)	0.162			0.011
Comorbidity burden	1.212 (0.261)	0.284			<0.001
Alcohol drinking	0.493 (0.684)	0.043			0.472
Functional status	1.485 (0.402)	0.229			<0.001
Perceived health status	−0.054 (0.026)	−0.137			0.039
Physical activity level	−1.835 (0.583)	−0.191			0.002
Block 4			0.370	0.057	<0.001
The presence of MCI	0.686 (0.543)	0.080			0.209
Depressive symptoms	0.356 (0.083)	0.303			<0.001
Model 2: MCI group (*n* = 127)			0.385	0.385	<0.001
Age	0.062 (0.052)	0.103			0.240
Female	1.587 (0.872)	0.162			0.071
Income	−0.638 (0.574)	−0.101			0.269
Education	−0.429 (0.380)	0.097			0.262
Being single	−0.656 (0.411)	−0.131			0.141
Living alone	−0.855 (0.581)	−0.117			0.144
Number of stressful life events	−0.121 (0.521)	−0.018			0.816
Comorbidity burden	0.965 (0.377)	0.215			0.012
Alcohol drinking	0.167(0.129)	0.098			0.198
Functional status	0.817 (0.491)	0.143			0.099
Perceived health status	−0.028 (0.036)	−0.068			0.429
Physical activity level	−2.245 (0.793)	−0.218			0.005
Depressive symptoms	0.317 (0.096)	0.297			0.001
Model 3: control group (*n* = 92)			0.352	0.352	<0.001
Age	0.029 (0.051)	0.061			0.570
Female	−0.199 (0.993)	−0.024			0.842
Income	0.151(0.518)	0.032			0.771
Education	−0.015 (0.459)	−0.004			0.973
Being single	0.314 (0.507)	−0.069			0.538
Living alone	−0.332 (0.753)	−0.048			0.660
Number of stressful life events	−0.857 (0.708)	−0.126			0.230
Comorbidity burden	1.179 (0.364)	0.361			0.002
Alcohol drinking	−0.068 (0.138)	−0.048			0.625
Functional status	0.174 (10.092)	0.019			0.874
Perceived health status	−0.011 (0.041)	−0.031			0.797
Physical activity level	−1.562 (0.821)	−0.185			0.061
Depressive symptoms	0.459 (0.205)	0.264			0.028

Note: Block 1: including social-demographic candidate correlates (age, gender, income, education, marital status, residence, stressful life events); B: unstandardized coefficient. SE: standard error; Beta: standardized coefficient.
